# Forecasting the incidence of salmonellosis in seniors in Canada: A trend analysis and the potential impact of the demographic shift

**DOI:** 10.1371/journal.pone.0208124

**Published:** 2018-11-27

**Authors:** Patricia Turgeon, Victoria Ng, Regan Murray, Andrea Nesbitt

**Affiliations:** 1 National Microbiology Laboratory, Public Health Agency of Canada, Saint-Hyacinthe, Canada; 2 Groupe de recherche en épidémiologie des zoonoses et santé publique, Faculté de médecine vétérinaire, Université de Montréal, Saint-Hyacinthe, Canada; 3 National Microbiology Laboratory, Public Health Agency of Canada, Guelph, Canada; 4 Centre for Food-borne, Environmental and Zoonotic Infectious Diseases, Public Health Agency of Canada, Guelph, Canada; Australian National University, AUSTRALIA

## Abstract

*Salmonella* infections remain an important public health issue in Canada and worldwide. Although the majority of *Salmonella* cases are self-limiting, some will lead to severe symptoms and occasionally severe invasive infections, especially in vulnerable populations such as seniors. This study was performed to assess temporal trends of *Salmonella* cases in seniors over 15 years (2014–2028) and assess possible impact of demographic shift on national incidence; taking into account of trends in other age groups. The numbers of reported *Salmonella* cases in seniors (60 years and over) in eight provinces and territories for a period of fifteen years were analysed (1998–2013) using a time-adjusted Poisson regression model. With the demographic changes predicted in the age-structure of the population and in the absence of any targeted interventions, our analysis showed the incidence of *Salmonella* cases in seniors could increase by 16% by 2028 and the multi-provincial incidence could increase by 5.3%. As a result, the age distribution amongst the *Salmonella* cases is expected to change with a higher proportion of cases in seniors and a smaller proportion in children (0–4 years old). Over the next decades, cases of infection, hospitalizations and deaths associated with *Salmonella* in seniors could represent a challenge to public health due to an aging population in Canada. As life expectancy increases in Canada, identification of unique risk factors and targeted prevention in seniors should be pursued to reduce the impact of the demographic shift on disease incidence.

## Introduction

In Canada, *Salmonella* is one of the leading causes of enteric illnesses with approximately 100 000 domestically acquired cases each year [1]. Of these, 80% are estimated to be of foodborne origin[1]. *Salmonella* also causes a higher proportion of hospitalisations associated with enteric illnesses than any other enteric bacteria annually, with 35% of hospitalisations related to domestically-acquired foodborne bacterial illnesses in Canada each year [1]. Although most infections of *Salmonella* are self-limiting, some people will experience severe symptoms and occasionally severe invasive infections. Some groups of individuals are considered more at risk for severe *Salmonella* infection, including young children, pregnant women, immunocompromised individuals and seniors. Among those groups, seniors (those 60 years and over) are one sub-population that is expected to change significantly in the next decade. As with many industrialized countries, the Canadian population is experiencing a demographic shift toward a larger population of seniors. Statistics Canada expects that the proportion of people aged 60 and over, which made up 20% of the Canadian population in 2013, will increase to almost 30% by 2028, representing an increase of more than 5 million people [2].

The aging Canadian population and the associated increases in public spending on health care could represent an important challenge. Although seniors represented 23% of reported *Salmonella* cases in adults in Canada, almost 50% of *Salmonella*-related hospitalizations and most of the deaths occurred in this age group [3]. The burden associated with *Salmonella*-related hospitalization is greater as people get older and seniors tend to stay longer in the hospital than other adults [4]. This prolonged stay, especially of older people who tend to be affected by more than one medical condition, may contribute to an increased cost to the health care system. In regards to this challenge, informed policy making and planning necessitates an understanding of the present and anticipated future trends of disease incidence. Knowledge on trends and changes over time could also help to understand transmission pathways for this specific age group. Therefore, the objectives of this study were threefold: 1-describe temporal trends from 1999 to 2013 of *Salmonella* cases in seniors; 2- assess the possible impact of demographic shift on multi-province incidence in 15 years (2014–2028); 3- use outbreak-related case data from one province to assess the impact of outbreak-related cases on temporal trends.

## Methods

### Data sources

#### National reported cases

In Canada, salmonellosis infections are reportable diseases and cases are captured from the provincial and territorial public health authorities/jurisdictions by the Public Health Agency of Canada's Canadian Notifiable Disease Surveillance System (CNDSS) [5]. Only laboratory confirmed cases are notified, that is isolation of *Salmonella* spp. (excluding *Salmonella* Typhi) from an appropriate clinical specimen. Depending on the province, the age of individuals is reported by aggregated age groups or specific age. For some provinces (representing 10% of the Canadian population) cases are currently aggregated yearly before being nationally reported. Due to these limitations, only provinces and territories reporting case level data (specific age and episode date) were considered for this study, and included British Columbia, Alberta, Saskatchewan, Ontario, Quebec, Newfoundland and Labrador and Yukon, representing 90% of the Canadian population and 85% of all the reported cases. For this study, the number of reported cases of *Salmonella* spp. was obtained from the CNDSS database from January 1, 1999 to December 31, 2013. Cases were broken down into four age groups: 0–4 years, 5–19 years, 20–59 years and 60 years and over (seniors). The age group of seniors was further divided into two sub-groups: people aged 60 to 79 years and people aged 80 years and over. Cases were then aggregated monthly for trend analysis. Aggregation by month was chosen to ensure a sufficient number of cases over the time period for analysis.

#### Ontario outbreak-related cases

The province of Ontario is the most populous Canadian jurisdiction, representing almost 45% of the national population and 46% of the reported cases of salmonellosis. In Ontario, salmonellosis is a reportable disease and all laboratory-confirmed cases are reported to local public health units. Investigations are performed for each case and findings (including outbreak information) are entered into the integrated Public Health Information System (iPHIS) database of the Ontario Ministry of Health and Long-Term Care [6]. Provincial confirmed *Salmonella* cases associated with an outbreak were aggregated by month, year, and age groups and were acquired from iPHIS from January 1^st^ 2005 to December 31, 2013. Prior to the implementation of iPHIS in 2005, outbreak and sporadic cases could not be differentiated.

#### Population at risk

Intercensal estimates or census data were used to calculate the appropriate population at risk and denominators for rate calculations from 1999 to 2013 for each age group [2]. Projected population estimates based on a medium growth scenario from Statistics Canada were used for population at risk and rate calculations for years 2014 to 2028 for each age group [2].

### Statistical analysis

For this study, temporal trends by age group were examined and descriptive analyses were performed. Descriptive and statistical analyses were performed using Microsoft Excel 2000 (Microsoft Corporation, Redmond, WA, USA) and STATA IC for Windows, version 14 (Stata Corporation, College Station, TX, USA). Statistical difference between incidences in age groups was assessed by calculating incidence rate ratio and the 95% confidence intervals associated.

#### Temporal trends in seniors

To assess temporal trends in the senior population, monthly counts of cases were regressed against time (January 1999 (month 1) to December 2013 (month 180)) using a Poisson model for every age groups (60 and over, 61–79, 80 and over) and the population at risk was included in the models as an offset variable. Temporal trend of cases over the time series was assessed and the period best describing the data in terms of periodicity was assessed by a periodogram using the epigram macro in Stata and was determined as a 12-month period. To account for annual periodicity (seasonality) of salmonellosis a sine and a cosine term were thus added to the models:

sin12=sin(2*π*month/12)

cos12=cos(2*π*month/12)

From the models, the effect of time (trend) was expressed as an incidence rate ratio (IRR) with 95% confidence intervals, IRR over one represents the monthly increase of incidence over the fifteen years of the study. Normality and stationarity of residuals were assessed graphically. Moreover, normality was assessed by a Shapiro-Wilk test, a p-value <0.05 indicating a non-normal distribution and stationarity of residuals was assessed by regressing them over time, a p-value <0.05 indicating an absence of a trend among residuals over time. Autocorrelations in residuals were assessed by a correlogram and a partial correlogram with 95% confidence intervals. To estimate the month where there was the most significance change in salmonellosis cases across the years, a Bayesian change point analysis using a Poisson distribution was performed. Non informative priors for the parameters were applied (flat priors for the means and a uniform on month 1–180 of the time period for the change point). The ratio with 95% confidence intervals between the two means was also estimated to assess the increase after the change point.

#### Expected number of cases

The model was then used to calculate monthly expected cases and incidence for the years 2014 to 2028 with population at risk being the projections of population from Statistics Canada. Expected numbers of cases for each age group were summed up to calculate the multi-jurisdictions expected incidence rate for 2028. This expected incidence was then compared to the average annual multi-jurisdictions incidence of the three last years of the study (2011 to 2013).

#### Case-study with outbreak-related data

Descriptive and statistical analyses were performed on outbreak-related data in Ontario. To assess the impact of outbreak-related data on the temporal trends of *Salmonella* infections assessed with previous models, two regression models were built from the Ontario outbreak-related data. *Salmonella* cases occurring in Ontario were extracted from the national database and: 1) Monthly counts of all *Salmonella* cases were regressed against time (month) using a Poisson model for every age group, without controlling for the number of cases related to an outbreak, and 2) Monthly counts of all *Salmonella* cases were regressed against time (month) using a Poisson model for every age group, and the number of outbreak-related cases was added as a covariate. For both models, population at risk was added to the models as an offset variable and a sine and cosine curve representing a 12-month period were added to account for annual periodicity. Differences between the goodness-of-fit of the models with and without controlling for outbreak-related cases were assessed by likelihood ratio tests. Z-tests were used to assess if there were statistically significant differences between the IRRs of the time variable, whether or not we adjusted for outbreak-related cases in statistical models.

## Results

### Number of cases and rates

From January 1999 to December 2013 in the provinces included in this study, 12 661 *Salmonella* cases were recorded in seniors (60 and over), resulting in a mean annual incidence rate of 15.45 cases per 100,000 persons. This incidence is not statistically different than the incidence for other adults (20–59 years old) (16.14 cases per 100 000 persons), but is lower than the incidence in children (54.53/100 000 for 0–4 years old and 18.74/10,000 for 5–19 years old). Mean annual cases and incidence for seniors and other age groups are presented in Table 1.

**Table 1:** Mean annual *Salmonella* cases and incidence (per 100, 000) for all age groups (1999–2013).

Age groups	Total number of cases	Annual mean	% of total number of cases	Multi-jurisdictional population	% of Total	Average annual incidence
**0–4**	13 304	887	16.3	1 626 564	5.5	54.5
**5–19**	15 495	1 033	18.9	5 511 734	18.6	18.7
**20–59**	40 143	2 676	49.2	16 579 000	56.1	16.1
**60+**	12 661	842	15.5	5 450 490	18.4	15.4
**60–79**	10 153	677	12.4	4 427 569	14.9	15.3
**80+**	2 508	167	3.1	1 022 921	3.5	16.3
**Total**	81 603	5 440		29 565 988		18.4

### Temporal trends in seniors

**Fig 1:**
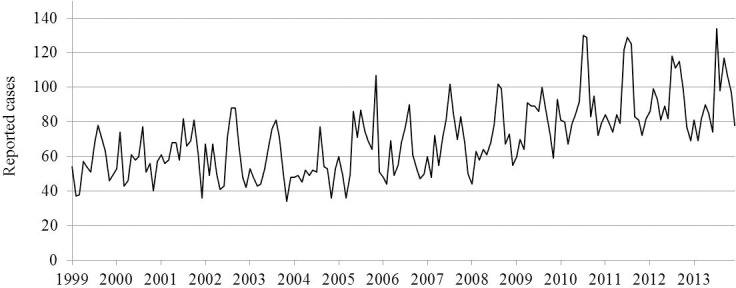
Reported *Salmonella* cases in seniors by month from 1999 to 2013 in Canada (Canadian Notifiable Disease Surveillance System).

According to the Poisson regression model over the 15 years of the study, the time variable (month) was associated with an IRR of 1.00097 (95% CI: 1.0006; 1.0013) for the years of the study, meaning a monthly average increase of 0.097%. The Shapiro-Wilk test indicated that residuals followed a normal distribution (p = 0.13), the regression of residuals over time attested the stationarity of the residuals (p = 0.07), and the correlogram and a partial correlogram did not reveal significant autocorrelation. The graph of the cases over time showed a non-constant increase over the 15 years of the study (Fig 1). From the Bayesian change point analysis, the change point is estimated to have occurred in 2005 and the mean number of cases increased by a factor of about 0.71 (95% CI: 0.68; 0.73) from 2005 to 2013.

### Expected numbers of cases

The average incidence (2011–2013) and the expected incidence rate, calculated from regression models for the years 2014–2028, are presented by age groups in Table 2. Overall an increase of 5.3% (95% CI = 1.1; 9.8) between the average annual incidence of the three last year of this study (2011 to 2013) and incidence in 2028 is expected, representing an increase of almost than 1,000 cases annually. Counter to the multi-jurisdiction incidence trend, incidence in children aged from 0 to 4 years old is expected to decrease by 29% (95% CI = 26.5;34.2). No statistically significant changes are expected in incidence rates in the age group of 5 to 19 years old and adults aged from 20 to 59 years old, with percentages of variation of -4.8% (95% CI = -9.4;0) and 3.6% (95% CI = -0.6;7.6), respectively. Graph of the cases over time (1999 to 2013) for those age groups are included in supporting information (S1–S3 Figs). In seniors, the incidence is expected to increase by 16.6% (95% CI = 9.9; 23.1). When looking at the sub-groups of seniors, incidence is expected to increase by 18% (95% CI = 10.7; 27.7) for the seniors aged from 60 to 79 years old and by 43% (95% CI = 23.7; 63.2) for seniors aged of 80 years and over. As a result, the age distribution among the *Salmonella* cases is expected to change with a higher proportion of cases aged of 60 to 79 years and also of 80 years and over, and with a decrease in cases aged of 0 to 4 years old (Table 3).

**Table 2:** Average *Salmonella* incidence rates (per 100, 000) for the years 2011 to 2013 and expected Salmonella incidence rate for 2028.

Age groups	Average incidence (2011–2013)	Expected incidence in 2028	CI 95%	Percentage of variation*
**0–4**	50.5	35.2	(33.3;37.1)	-29
**5–19**	20.1	19.1	(18.2;20.1)	-4.8
**20–59**	16.9	17.5	(16.8;18.2)	3.6
**60+**	16.9	19.7	(18.6;20.8)	16.6
**60–79**	15.9	18.8	(17.6;20.3)	18
**80+**	17.7	25.3	(21.9;28.9)	43
**Total**	18.4	19.4	(18.6;20.2)	5.3

* This is the variation between the average incidence (2011–2013) and the expected incidence in 2028. For example, for age group 0–4 years: ((35.2–50.5)/50.5) *100

**Table 3:** Annual number of *Salmonella* cases and percent of cases by age for the years 2011 to 2013 (average) and 2028 (expected).

Age groups	Average cases (2011–2013)	% of all cases	Expected cases (2028)	CI 95%	% of all cases	CI 95%
**0–4**	877	14.7	671	(633;709)	9.7	(9; 10.4)
**5–19**	1092	18.3	1161	(1105;1217)	16.8	(15.9;17.7)
**20–59**	2882	48.4	3277	(3174;3380)	47.4	(46.3;48.6)
**60+**	1108	18.6	1798	(1684;1912)	26.0	(25;27.1)
**61–79**	855	14.3	1269	(1170;1386)	18.4	(17.5;19.3)
**80+**	253	4.2	529	(468;590)	7.7	(7,8.3)
**Total**	5959		6907	(6596;7218)		

### Case-study with outbreak-related data

**Fig 2:**
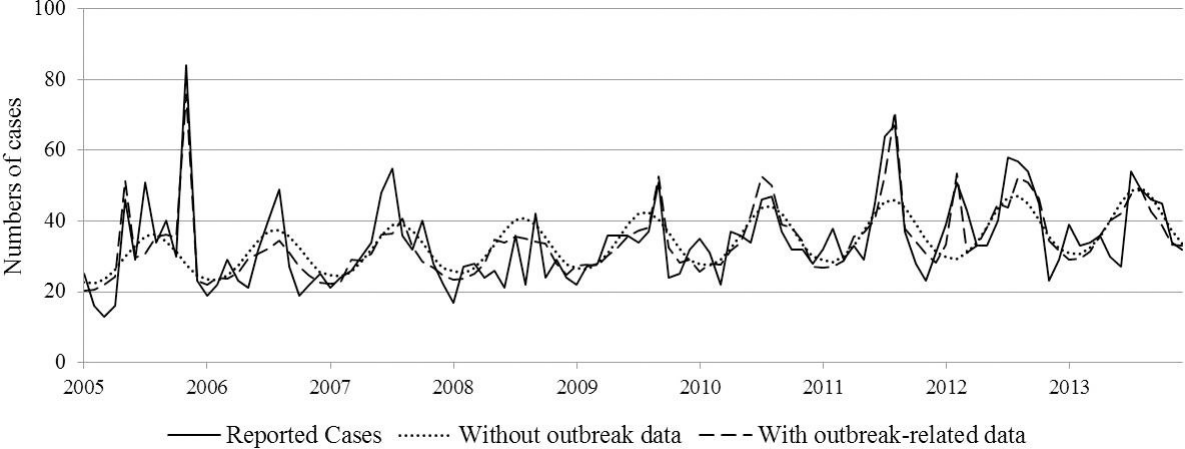
Reported *Salmonella* cases in Ontario and predicted cases from models with and without outbreak-related covariate (iPHIS).

From 2005 to 2013, 8.1% of reported *Salmonella* cases in Ontario were associated with an outbreak. No statistically significant differences in outbreak cases were observed amongst percentages of cases by age groups by month and by years. When adjusting regression models with the number of cases related to an outbreak as a covariate, predicted numbers of cases fitted the peaks of the data better than data for all cases by age groups as observed with the model in seniors (Fig 2). Nonetheless, the likelihood ratio tests indicate that models adjusted for outbreak-related cases did not have a better goodness-of-fit (P-values <0.001, for all age groups). Moreover, when comparing the IRRs of the time variable, there were no statistically significant differences between the IRRs, whether or not we adjusted for outbreak-related cases in statistical models.

## Discussion

The first objective of this study was to describe temporal trends from 1999 to 2013 of *Salmonella* cases in seniors. The analyses revealed a significant increase in the incidence rate over the study period in seniors. An American study also reported an increase in the rate of salmonellosis in older adults (aged ≥65 years) from 1996 to 2012, without explanation for this result [7]. In our study, the most significant increase was seen in 2005. An important outbreak of *Salmonella* Enteritidis occurred in the fall of 2005 in Ontario, with almost 552 laboratory confirmed cases (60 in seniors), which can explain the sharp increase in cases for this year at the multi-jurisdiction level [8]. However, the increase in the incidence rate in seniors remained significant even after this outbreak. While the increase in incidence of reported *Salmonella* cases in seniors cannot be fully understood, this increase could be associated with an increase of the prevalence of a specific serotype of *Salmonella* in products for which seniors are more susceptible or more exposed to. Since 1999, the number of reported human cases of *Salmonella* Enteritidis reported to the Public Health Agency of Canada’s National Enteric Surveillance Program (NESP) has increased, nearly tripling, and the proportion of this serotype of all the *Salmonella* serotypes increased from 14% in 1999 to 32% in 2013 [9]. Demographic data such as age are not captured with the serotype information at the national level, however data from FoodNet Canada, the Canadian integrated sentinel site surveillance program, show from 2006 onwards that approximately 40% of *Salmonella* in 60 years and over is from *Salmonella* Enteritidis each year (FoodNet unpublished data). Poultry and eggs have been identified as important exposure sources of *Salmonella* Enteritidis for humans and survey on seniors have found that a non-negligible proportion of older adult do not follow recommended food safety practices associated with raw or runny eggs consumption and cooking and handling raw poultry [10–16]. A combination of an increased presence of *Salmonella* Enteritidis in eggs and poultry commodities and risky food preparation behaviors among senior may have contributed to the increase of the incidence of salmonellosis, but more investigation and data are needed to explore this relationship.

*Salmonella* infections are also associated with travel outside Canada [14, 15]. Changes in travel habits in seniors over the last decade could have influenced the incidence rate of salmonellosis in this age group. However, no substantial changes in the proportion of cases associated with travel in seniors were recorded by FoodNet Canada, from 2006 to 2013 [17]. S*almonella* has also been recognized to be a causative agent of outbreaks in nursing homes and long-term care facilities [18, 19]. Demographic data associated with outbreak-related cases are not captured in a standardized or systematic manner across the country. However, from the Ontario iPHIS database, no statistically significant differences in the proportion of outbreak cases were observed amongst seniors during the study period in Ontario. This stable proportion suggests that effect of outbreaks on the incidence in seniors over the years could be negligible. There is no indication that outbreak patterns in seniors would differ in other jurisdictions outside Ontario.

Findings from this study also showed that the incidence of laboratory-confirmed *Salmonella* infection is similar in the senior age group and younger adults. This is consistent with data from other industrialized countries that show peaks in childhood rate and similar rate in adult age groups [7, 20] Nonetheless, salmonellosis in seniors is often associated with more severe consequences than younger adults [21–23].

The second objective of the study was to assess the possible impact of demographic shift on multi-jurisdiction incidence in 15 years. Based on our results, the multi-provincial incidence is expected to increase by 5.3% by 2028 and the age distribution amongst the *Salmonella* cases is expected to change with a higher proportion of cases aged 60 years and over. The number of cases and proportion of cases over 80 years old could almost double. This result is noteworthy, because this could represent an important challenge, as higher hospitalization and death rates have been reported for seniors, impacting the health care system in terms of capacity and expenditures. The economic burden of *Salmonella* is substantial and hospitalizations and deaths associated to severe infections are reported to account for much of this cost (7% and 90%, respectively) [24]. Because of their vulnerable status, identification of unique risk factors (including specific exposure and behavioural factor) and targeted prevention with seniors should be pursued, especially for those aged 80 and over. More data on serotypes affecting seniors could also help to better understand the transmission pathways and the elaboration of targeted measures for this age group. Those measures could contribute to reduce the impact of demographic change on the number of cases, hospitalizations and deaths associated with salmonellosis.

Some limitations should be taken into account when looking at the results of this study. Although *Salmonella* is a notifiable disease in Canada, cases could have been under estimated in reportable disease databases. According to a study on burden of disease of foodborne illness in Canada, for every case of *Salmonella* infection reported in the CNDSS, there were an estimated 26 cases annually in the Canadian population, when accounting for under-reporting and under-diagnosis [25–27]. Nonetheless, there is no indication of change over time regarding this difference in rate of medical care-seeking, suggesting that this difference would not have affected the trend over the study period and the change in the age distribution amongst the *Salmonella* cases expected for the next 15 years. Changes in case ascertainment or in diagnostic methods could have influenced the trend observed, however changes would have influenced all the age groups in the same way. Nonetheless, there were no substantial changes to the case definition, diagnostic testing and reporting practices during the study period. We should also recognize some limitation regarding the methods. Expected cases were calculated from models that did not fit the data perfectly. However, since the objective was to assess the trend over the year and not predict the exact number of cases at a specific month, we are confident that this objective was met even with an imperfect model fit.

Most *Salmonella* infections are sporadic cases, but some are part of outbreaks. In our study, outbreak and sporadic cases could not be differentiated at the multi-jurisdictional level. Trends and expected changes in incidence were thus calculated with the effect of outbreaks in the models. Although models visually showed better adjustment to the data when adding the number of outbreak-related cases as a covariate with the Ontario data, the goodness-of-fit and the trends over the study period did not change significantly with or without controlling for outbreak-related cases. It is unknown whether this non-significant difference was due to a lack of statistical power or to a true absence of effect but because outbreaks were spread out across the time period, it is unlikely that controlling for outbreaks in the multi-jurisdictional models would have produced significantly different results.

Our results indicate an expected increase in salmonellosis in seniors while there will be an expected decrease in children. With the demographic changes predicted in the age-structure of the population, there will be an increase in the overall incidence. However, this approach focused on a change in a single factor (age-structure of the population) and was intended as a scoping exercise. Some other aspects that could change the age-specific probabilities of disease in the future were not considered in this analysis. Such factors may include health care availability, extended aging in a residence home, and change in the incidence of chronic diseases, which could affect the vulnerability of some age groups for infectious diseases [28–30]. Moreover, the unusual change in *Salmonella* Enteritidis incidence over the study period may have heavily influenced the predictions. Prevention measures, food safety interventions and public health initiatives, specifically for *Salmonella* Enteritidis, could also impact the incidence of salmonellosis in future [12]. *Salmonella* is one of the most prevalent bacteria associated with enteric illness in Canada and over the next decades, cases of infection and resulting hospitalizations and deaths associated with *Salmonella* in seniors is expected to increase due to an aging population in Canada.

## Supporting information

S1 Fig Reported *Salmonella* cases in the age group of 0 to 4 years by month from 1999 to 2013 in Canada (Canadian Notifiable Disease Surveillance System). (TIF)

S2 Fig Reported *Salmonella* cases in the age group of 5 to 19 years by month from 1999 to 2013 in Canada (Canadian Notifiable Disease Surveillance System). (TIF)

S3 Fig Reported *Salmonella* cases in the age group of 20 to 59 years by month from 1999 to 2013 in Canada (Canadian Notifiable Disease Surveillance System). (TIF)
