# Supplementary figures and images for: Forecasting the incidence of salmonellosis in seniors in Canada: A trend analysis and the potential impact of the demographic shift
Source: PLoS One. 2018 Nov 27;13(11):e0208124. doi: 10.1371/journal.pone.0208124 (PMC6258544; doi:10.1371/journal.pone.0208124)

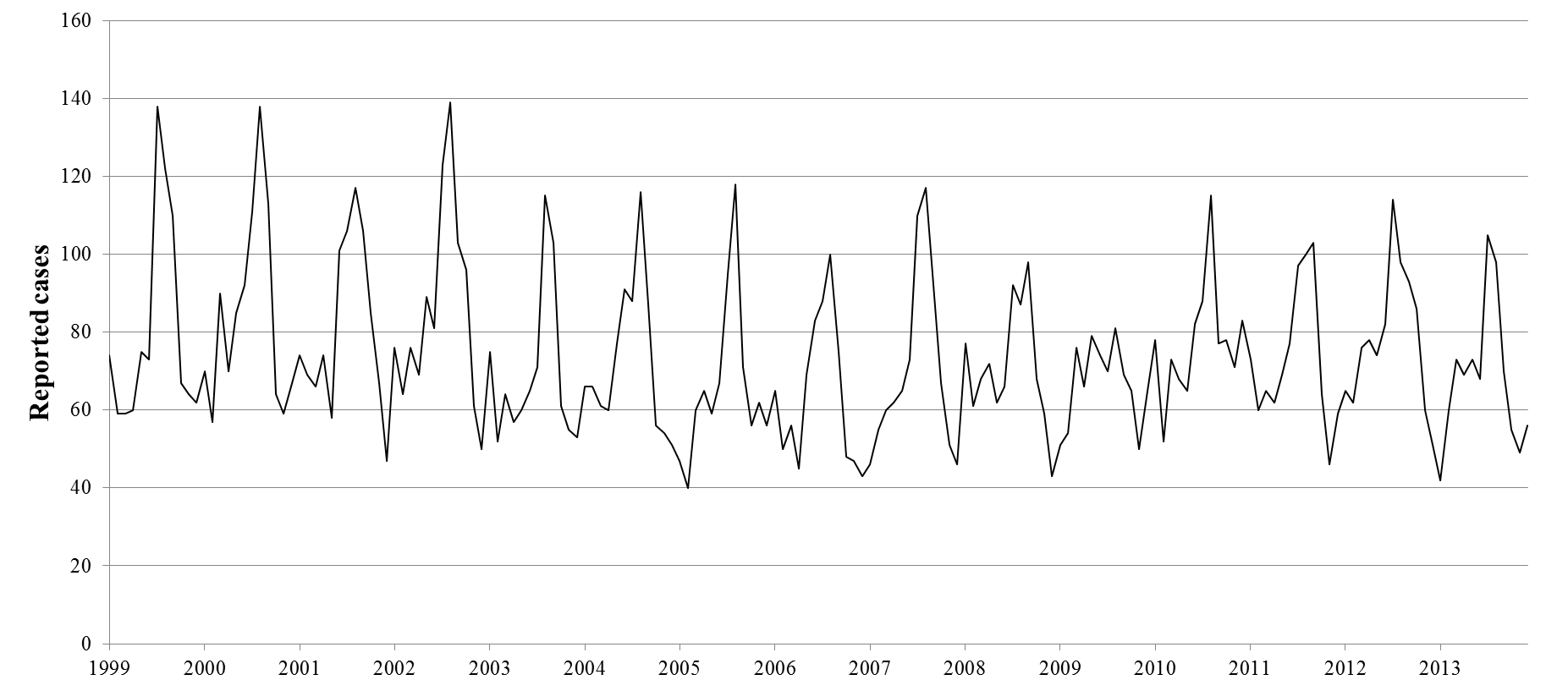

Supplement: S1 Fig — (TIF) [file pone.0208124.s001.tif]

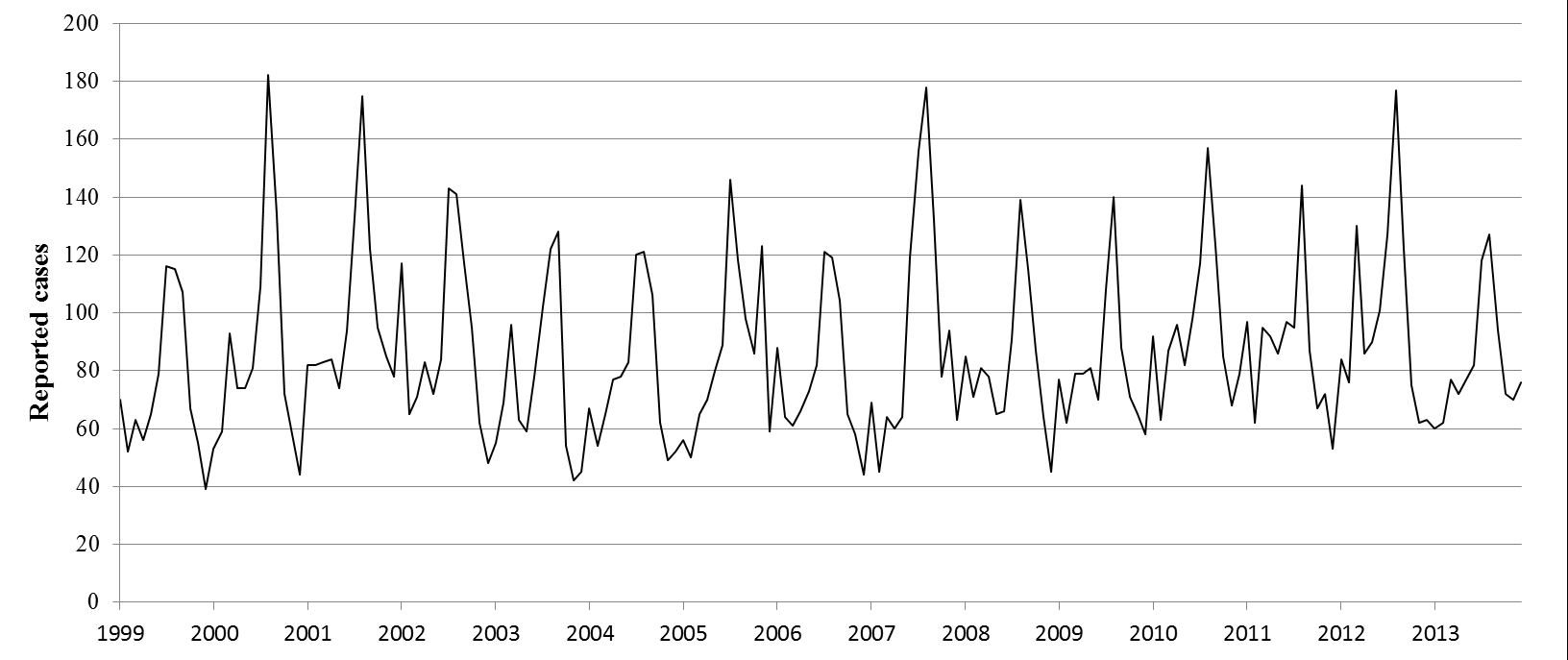

Supplement: S2 Fig — (TIF) [file pone.0208124.s002.tif]

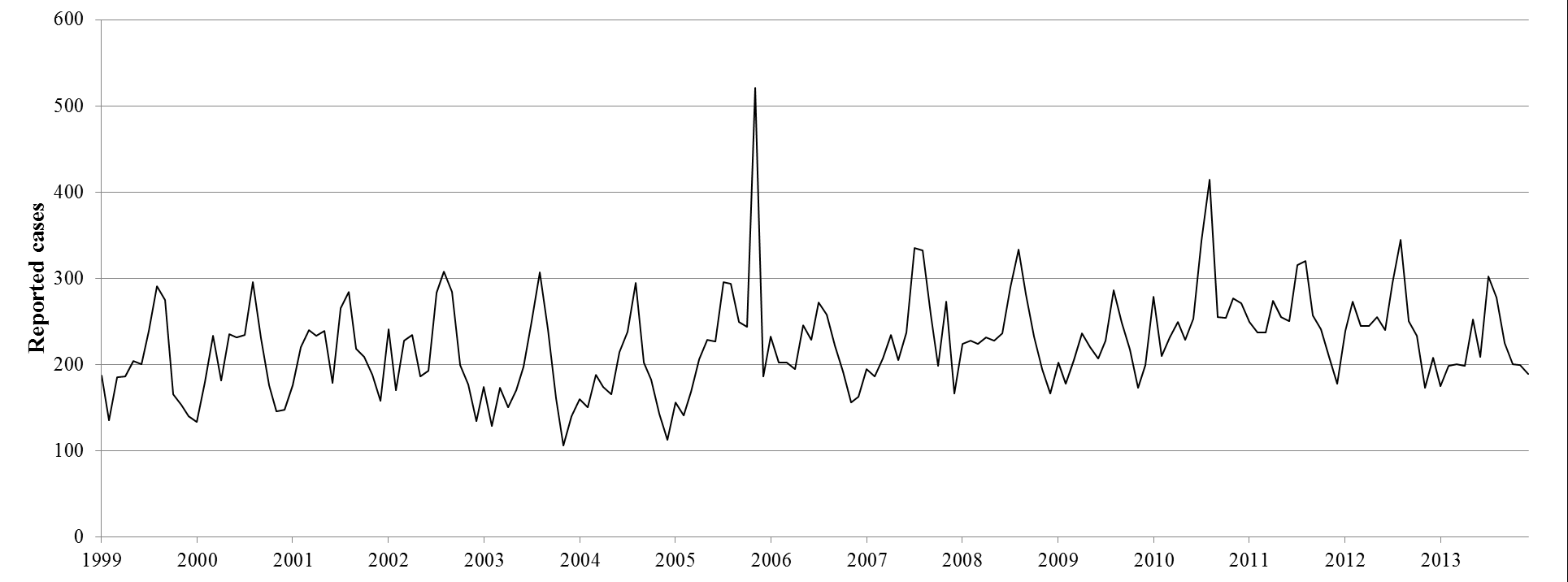

Supplement: S3 Fig — (TIF) [file pone.0208124.s003.tif]
